# Matching Rheology, Conductivity and Joule Effect in PU/CNT Nanocomposites

**DOI:** 10.3390/polym13060950

**Published:** 2021-03-19

**Authors:** Leire Sangroniz, Maite Landa, Mercedes Fernández, Antxon Santamaria

**Affiliations:** 1 POLYMAT and Department of Polymers and Advanced Materials: Physics, Chemistry and Technology, Faculty of Chemistry, University of the Basque Country UPV/EHU, Paseo Manuel de Lardizábal, 3, 20018 Donostia-San Sebastián, Spain; leire.sangroniz@ehu.eus (L.S.); mercedes.fernandez@ehu.eus (M.F.); 2 FIBER PROFIL, Calle Bikuña Almirantea, 27, 20230 Legazpi, Gipuzkoa, Spain; mlanda@fiberprofil.com

**Keywords:** Joule effect, nanocomposites, stimulus-responsive, hot melt adhesives, PU, CNT

## Abstract

We investigated polyurethane (PU)–carbon nanotube (CNT) nanocomposites (PU/CNT) in a range of concentrations from 1 to 8 wt% CNT as hot melt adhesives. We studied the thermal properties of the nanocomposites, which is relevant from an applied point of view. The phase angle plots versus complex modulus results revealed the existence of a maximum above a given CNT concentration. The intensity of the peak and associated relaxation time was analyzed with percolation theory, leading to a new method to determine the rheological percolation threshold. A lower threshold value was obtained from the electrical conductivity data, which was justified recalling that the hopping/tunnelling effect takes place in the nanocomposite, as stated by previous studies in the literature. Joule effect studies indicated that the heating effect was very significant, reaching temperature increases, ΔT, of 60 °C for low voltages. For the first time, the percolation equation was applied to the ΔT to obtain the corresponding threshold. Stimulus-responsive systems were conceived considering the correlation between the ΔT and the conductivity. The case of PU/CNT nanocomposites acting as hot melt adhesives that are welded/unglued by applying/removing an electrical voltage is presented.

## 1. Introduction

In recent decades, polymer nanocomposites have attracted attention due to their outstanding physical properties, such as mechanical performance and, especially, electrical performance, in the case of conductive nanoparticles [1,2]. Taking advantage of the semiconductor feature of polymer nanocomposites, stimuli responsive materials have been developed [3,4]; applying physical stimuli such as light [5], electric current [6,7], magnetic field [8] or a variation in the pH, temperature, shape or solubility [9] of the material can be changed in a controlled way.

Polyurethanes (PU) are interesting polymer matrices for nanocomposites, in particular those that are used as hot melt adhesives due to microphase separation and crystallization. The basic requirements of hot melt adhesives are to be tacky in the molten state and to crystallize upon cooling as room temperature is reached to produce a permanent weld. Years ago, researchers developed semiconductive nanocomposite adhesives based on PU and carbon nanotubes (CNT) that showed excellent tackiness and enhanced crystallization rates [10,11,12].

The semiconductive character of certain nanocomposites brings about new applications in the field of stimuli responsive materials. One case of physical response to an external stimulus is in applying an electric current, which results in an increase of temperature [13] due to the well-known Joule heating effect. In the literature, there are several works regarding applications of the Joule heating effect, such as de-icing polymer nanocomposite devices [14,15,16,17,18] and curing thermostable materials [19,20]. However, works focusing on the correlation between the heating effect and the content of nanoparticles, as well as on the relationship between the temperature increase and electrical conductivity, are scarce [17,19].

In this work, we investigated the feasibility of a series of PU/CNT nanocomposites in a wide range of CNT content to face to these shortages. Considering the hot melt application of these nanocomposites, the thermal properties of the nanocomposites are addressed. Both rheological and electric percolation are discussed focusing on the physical meaning of the network for each case and proposing a percolation threshold based on the temperature increase associated with the Joule heating effect. We performed a thorough study of this effect and its relationship with the electric conductivity opening routes for the elaboration of stimulus-responsive hot melt adhesives.

## 2. Materials and Methods

### 2.1. Materials and Preparation

The semi-crystalline polyurethane employed in this work was provided by Merquinsa (PB121, Montmeló, Spain) and was used as a hot melt adhesive. According to previous studies [10,11,12], the hard segment content (10%) was constituted by diphenylmethane diisocyanate and 1,4-butanediol, whereas the 90% soft segment was polycaprolactone. The melting of the crystalline phase took place at *T* = 54 °C and its crystallization at *T* = 27 °C. Multiwall carbon nanotubes from Cheap Tubes Inc. (Grafton, Burlington, VT, USA) with a diameter between 30 and 50 nm and a length of 10–20 μm were employed.

Nanocomposites were prepared in the molten state. First, PU and CNT were mixed in powder form, employing a Mill Retsch ZM 200 (Haan, Germany), and then a Haake Mini-Lab (ThermoFisher Scientific, Waltham, MA, USA) twin screw extruder, at 100 rpm and 115 °C for 4 min, was employed to obtain the final product. SEM images showed that the CNTs were homogeneously dispersed in the nanocomposite with the employed procedure.

### 2.2. Measurements

The thermal properties were analyzed through differential scanning calorimetry (DSC) employing PerkinElmer Pyris I equipment (PerkinElmer, Waltham, MA, USA). The thermal history of the samples was completely erased by keeping the sample at 120 °C for 10 min; after that, the sample was cooled down to 0 °C and heated again, employing a rate of 10 °C/min.

The samples were analyzed employing atomic force microscopy (AFM, Bruker/Veeco/Digital Instruments, Billerica, MA, USA). Polymer/acetone solutions were spread on a mica substrate using a spin coating technique. A Nanoscope IV multimode AFM controller was employed (Bruker/Veeco/Digital Instruments, Billerica, MA, USA) in tapping mode etched silicon probes (TESP).

The samples to be measured were obtained by hot pressing at 115 °C for 2 min without pressure to thermostatize the material and for 8 min under pressure (*p* = 150 bar); then the material was cooled down to room temperature. Rheological experiments were performed in an ARG2 rheometer (TA Instrument, New Castle, DE, USA) with a 12 mm diameter parallel plate geometry. Small amplitude oscillatory shear (SAOS) experiments were carried out at 100 °C in the linear viscoelastic zone covering a frequency range of 0.01 to 100 Hz.

The electric conductivity was measured using an ARES rheometer (TA Instrument, New Castle, DE, USA) coupled to a Novocontrol (Madrid, Spain) interface at a temperature of 20 °C, taking advantage of the dielectric analysis option, applying a voltage of 1 V in a frequency interval from 2 × 10^1^ to 10^6^ Hz. To compare the results with the rheological data, the conductivity was also measured in the molten state at 100 °C.

**Figure 1:**
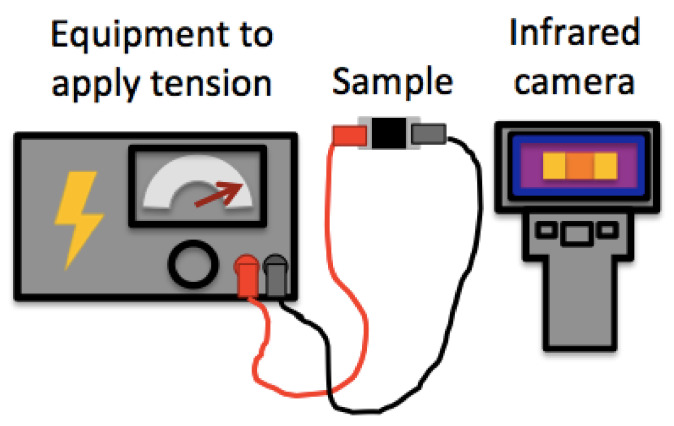
Schematic representation of the home-made equipment to analyze the Joule heating effect.

The Joule heating effect was studied employing an infrared camera (FLIR, Wilsonville, OR, USA) to measure the temperature using home-made equipment. The sample was connected to a voltage source that applied electric tension (Figure 1) using a compression molded sheet of 40 × 20 × 1 mm. Voltages between 10 and 50 volts were applied, and the average temperature of the sheet was measured. The intensity of the current for each applied voltage was also measured.

## 3. Results

### 3.1. Thermal Properties

Hot melt adhesives are tacky in the molten state, which allows for their application of on surfaces to be welded, but, when cooled down, they solidify, bringing about permanent welding. The PU/CNT nanocomposites studied in this work were semicrystalline, due to the presence of 90% PCL, which constitutes the soft segment. To study their potential use as hot melt adhesives, a thermal characterization is necessary. This is more relevant considering that relatively high temperatures (eventually above melting temperature) can be reached by means of the Joule heating effect.

**Figure 2:**
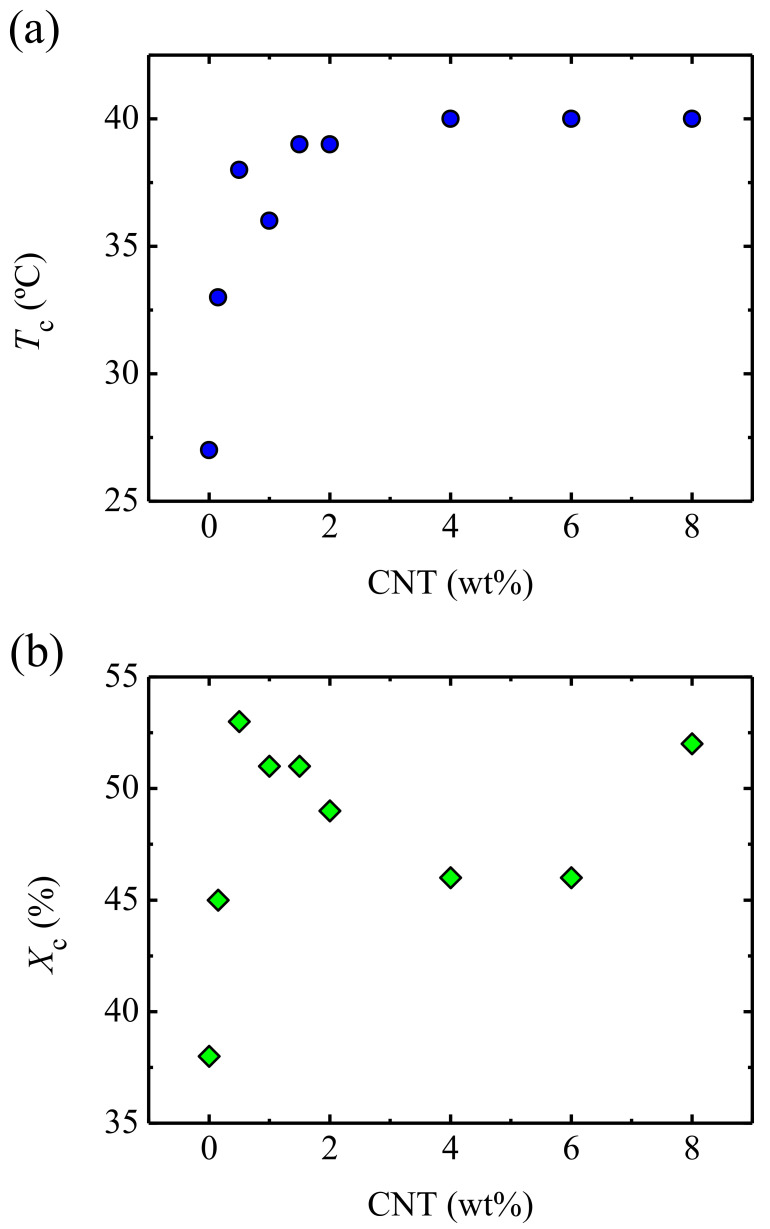
(**a**) Crystallization temperature and (**b**) crystallinity level for polyurethane (PU)-carbon nanotube (CNT) nanocomposites.

Dynamic thermal scans were performed to determine the melting and crystallization temperatures, as well as the crystallinity level, of the different samples. The study revealed that the melting temperature, *T*_m_, did not change significantly with the presence of CNTs: a value of *T*_m_ = 54 °C was obtained for neat PU, whereas for the nanocomposite with the highest CNT content (8 wt% CNT), the corresponding value was 56 °C. Therefore, very similar temperatures were required to reach the tacky state in both PU and PU/CNT nanocomposites. On the other hand, the crystallization temperature, *T*_c_, increased considerably with the presence of CNTs. This can be seen in Figure 2, which showed that the *T*_c_ jumped from *T*_c_ = 27 °C for PU to *T*_c_ = 40 °C when 8 wt% CNT was added. This was due to the nucleating effect of CNTs, as was reported in the literature [21,22,23,24,25,26,27]. Nucleation is discussed in more detail at the end of this section.

The effect of CNTs on the crystallinity level, displayed in Figure 2, was more complex. The crystallinity increased from 24% for neat PU to 53% for the nanocomposite, which contained 0.5 wt% CNT, and then decreased. Wu et al. [21] observed a similar behavior that was explained assuming a competition between the nucleating effect and restriction of chains mobility, which reduced the crystallization capability.

**Figure 3:**
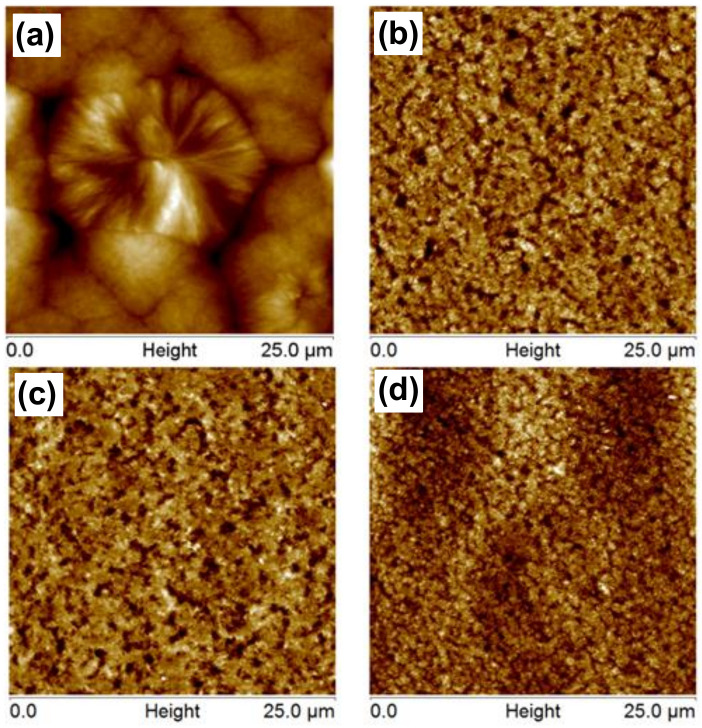
Atomic force microscopy (AFM) images obtained after melting the sample and cooling down for (**a**) PU, (**b**) 0.5 wt% CNT, (**c**) 2 wt% CNT and (**d**) 4 wt% CNT nanocomposites.

The nucleation effect of the carbon nanotubes was observed in the AFM results, as shown in Figure 3. The PU displayed a spherulitic-like structure with a diameter of approximately 20 μm. With increasing CNT content, the size of the morphological structure was reduced; this reduction was about 10 times for the nanocomposite with 4 wt% CNT. The presence of CNTs changes the morphology by modifying not only the size of the spherulites but also its structure, since an axialite-like morphology can be observed [23].

### 3.2. Rheological Properties

SAOS experiments are typically performed to characterize the viscoelastic properties of nanocomposites. If an appropriate dispersion is reached, the presence of nanoparticles above a certain concentration significantly affects the mobility of the polymer chains, which is evidenced at the low frequency zone equivalent to long experimental times, i.e., the terminal viscoelastic zone. Usually, the impact of nanoparticles on the shear elastic modulus, *G′*, as a function of frequency is investigated. We observed a *G′* levelling off, which reflects a solid-like behavior; this arises from the formation of a percolated network due to particle–polymer chain interactions. This network restricts the mobility of the chains as a whole, which is reflected in the response of terminal viscoelastic zone [28,29,30,31].

The phase angle *δ* (arc tan *G″*/*G′*) was also greatly affected by the presence of nanoparticles; however, this has been scarcely studied in the literature. To investigate this rheological parameter, a Mavridish-Shroff or Booij-Palmen plot [32,33,34], i.e., the phase angle as a function of a complex modulus G* ($\left|G^{*}\right|=\sqrt{G\prime^{2}+{G\prime \prime }^{2}})$, is particularly suitable. Typically, for a molten linear homopolymer, the phase angle has a value near 90° at low frequencies (low G* values) due to the very predominantly viscous character of the material (*G″* >> *G′*). As the frequency is increased, there is a transition from whole motion of the chain to segmental motion, owing to entanglements; this results in a rapid reduction of the phase angle as the rubbery zone (*G′* > *G″*) is reached [35].

A completely different behavior was observed for nanocomposites, since nanoparticles hinder polymer chain motion. The phase angle growth as frequency (or G*) was decreased, as expected for a linear polymer, which was not noticed for nanoparticle concentrations above a certain value. Instead, a maximum at intermediate frequencies was observed, denoting the transition from the short mobility restriction imposed by entanglements to the restriction impaired by the polymer/nanoparticle network [28,30,36,37]. We recall that the phase angle (arc tan *G″/G′*) stands for the dissipated energy associated with chain dynamics: The lower its value is, the higher the elasticity associated with mobility constraints is.

**Figure 4:**
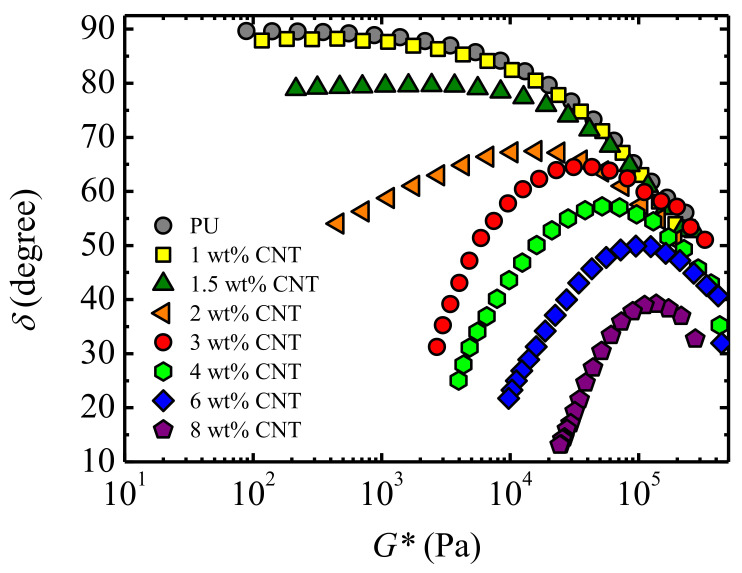
The phase angle as a function of the complex modulus for PU and PU/CNT nanocomposites measured at 100 °C.

As can be seen in Figure 4, a reduction of the phase angle, noticed at low G* or frequencies, was observed as the concentration of CNT nanoparticles was increased. For concentrations above 2 wt%, a maximum in the phase angle *δ* was observed. As the concentration increased, the peak decreased and was shifted to higher frequencies (higher G* values, Figure 4). Therefore, the mobility reduction associated with polymer–CNT interactions can be evaluated by the analysis of the *δ* peak.

**Figure 5:**
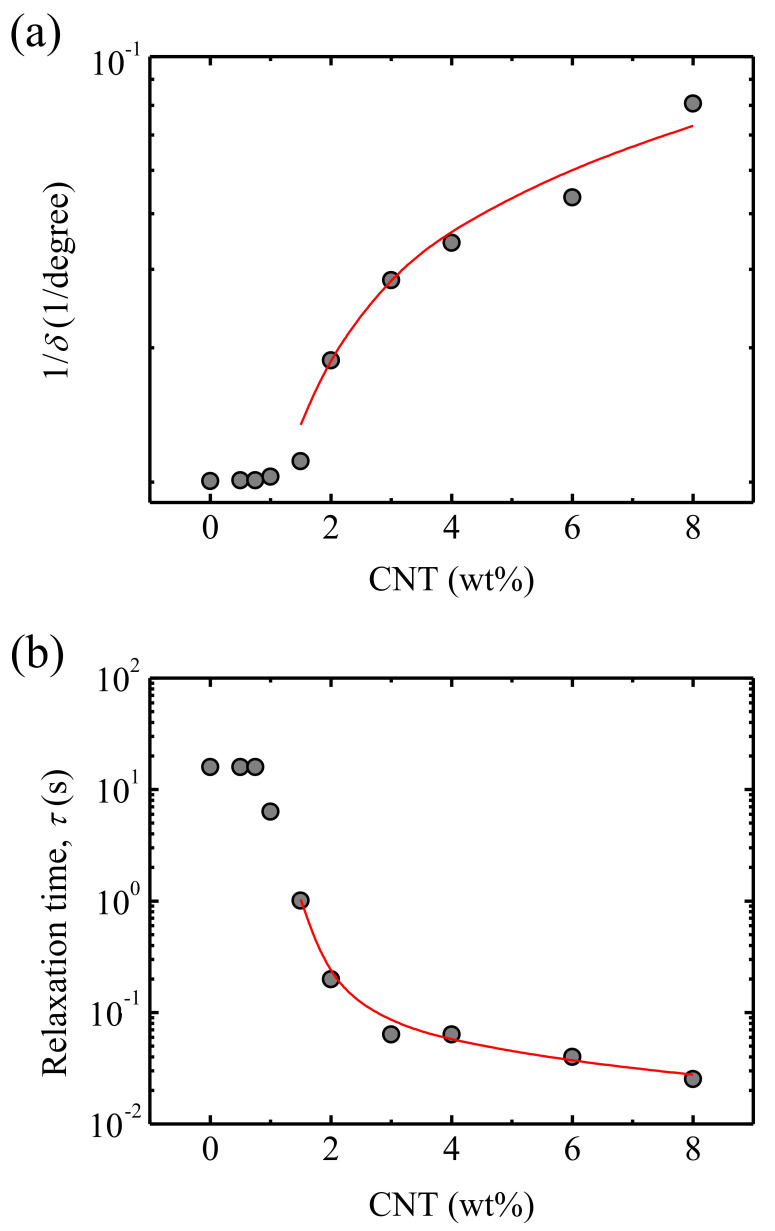
(**a**) Inverse of the phase angle corresponding to 0.01 Hz and (**b**) the relaxation time obtained from the inverse of the frequency of the maximum both as a function of the CNT wt%. The data are fitted to Equation (1), with *ϕ_c_* = 1 wt% CNT for both plots, and *t* = 0.64 and *t* = −0.64 for 1/*δ* and τ, respectively.

Taking advantage of this deduction, a new method to determine the concentration of nanoparticles necessary to form a three-dimensional percolated network, i.e., a percolation threshold, was proposed. For this, the inverse of the phase angle was employed; as explained before, for a molten polymer, the phase angle at low frequencies is close to 90°, which implies an inverse value, 1/*δ*, slightly higher than 1 × 10^−2^ 1/degree. These are the values that correspond to a viscous state, with no external impediment for chain diffusion. On the other hand, the phase angle decreased as CNT particles interacted with polymer chains owing to motion hindering, bringing about higher 1/*δ* values. Therefore, the level of motion hindrance can be expressed in terms of the inverse of the phase angle, 1/*δ*. In Figure 5a, the inverse of the phase angle at the lower frequency measured in SAOS experiments, 0.01 Hz, was plotted as a function of the CNT wt%. The figure suggests that, for concentrations above 1.5 wt%, the impediment to the mobility jumped one order of magnitude as a result of a qualitative change in the number of polymer/CNT interactions. The higher concentrations corresponded to the formation of a three dimensional percolated network, which practically cancels the mobility of the chains under the conditions of our experiments. The data of Figure 5a were fitted to the following equation, derived from the statistical percolation theory [38,39,40],

(1) $$x=x_{0}\;{\left(\phi -\phi_{c}\right)}^{t}$$

where *X* is the physical property under evaluation (in this particular case 1/*δ*), *ϕ* is the CNT concentration, *ϕ_c_* is the percolation threshold, and *t* is an adjustable parameter.

A rheological percolation threshold *ϕ_c_* = 1 wt% CNT was obtained, which means that above this concentration, a three dimensional network was formed, whereas below this concentration, the presence of nanoparticles was not enough to substantially alter the terminal viscoelastic zone. In fact, a qualitative change in the terminal zone was noted because only the nanocomposites with a CNT concentration above 1.5 wt% CNT showed a maximum in the MSVP (phase angle vs. G*) plots (Figure 4). The polymer matrix and the nanocomposites with a low CNT concentration showed a continuous increase of the phase angle as the frequency was reduced, since there was an enhancement of the mobility of the chains from the restrictions imposed by entanglements at short times or high frequencies [35].

Therefore, there was not any phase angle discontinuity for these systems. For concentrations above the percolation threshold, the phase angle increase observed with the lowering frequency was interrupted by the restriction imposed by the polymer/CNT network, thus giving rise to the maxima observed in Figure 4. Both the position and magnitude of the *δ* maximum changed with the CNT concentration, because the strength of the network increased with the density of the interactions. The time for the hindering effectiveness of the network was reduced as the CNT concentration increased.

This time is equivalent to the relaxation time, *τ*, which can be obtained from the inverse of the frequency at which the *δ* maximum takes place. As can be deduced from Figure 4, the corresponding relaxation times of the percolated nanocomposites decreased with an increasing amount of CNTs. This reflects that with the increasing CNT concentration, the impediment of the mobility of the chain occurred at lower time scales, and therefore shorter chain segments were affected or shorter distances between polymer/CNT interactions were produced. A corollary of this reasoning is the case of systems with no polymer/nanoparticle interactions in which the relaxation time would be infinite.

In Figure 5b, the relaxation time obtained from the inverse of the frequency corresponding to the *δ* maxima, observed in Figure 4, is plotted. The percolation equation used to fit the phase angle versus the CNT concentration (Equation (1), Figure 5a) fits well our relaxation time data taking a negative value of *t* (*t* = −0.64) and the same value for the percolation threshold, *ϕ_c_* = 1 wt% CNT, as in the analysis of 1/*δ*.

In the framework of our hypothesis, the physical meaning of *ϕ_c_* = 1 wt% CNT corresponds to the concentration value, which marks the minimum distance between polymer/CNT interactions to create a rheologically effective network.

### 3.3. Electrical Conductivity

**Figure 6:**
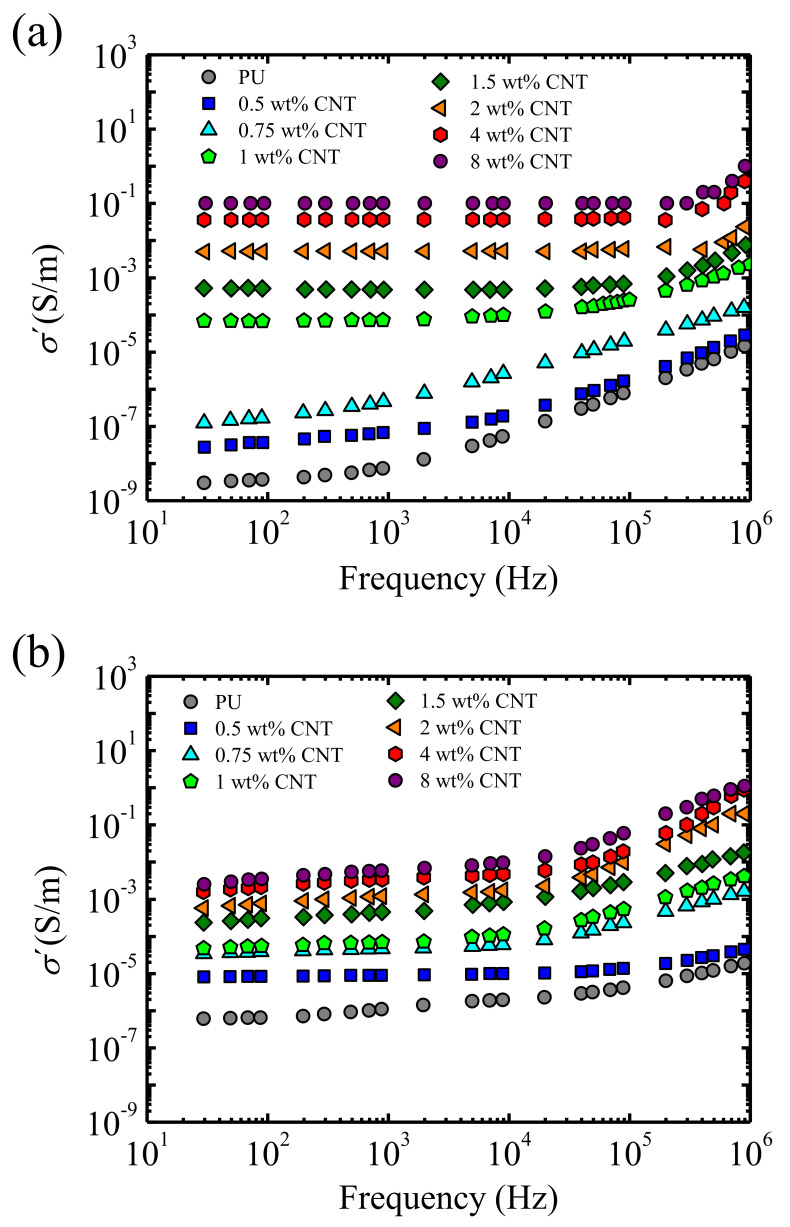
The electrical conductivity as a function of the frequency for the PU and PU/CNT nanocomposites measured at (**a**) 20 °C and (**b**) 100 °C.

The real part of the conductivity, $\sigma \prime =\varepsilon_{0}2\pi \varepsilon \prime \prime$ (*ε*_0_ vacuum permittivity, 8.85 × 10^−12^ Fm^−1^, and *ε″* imaginary part of the dielectric constant), as a function of frequency, was analyzed for the PU and PU/CNT nanocomposites. As can be observed in Figure 6, in the low frequency zone, the conductivity levelled off at 20 °C and tended to level off at 100 °C.

**Figure 7:**
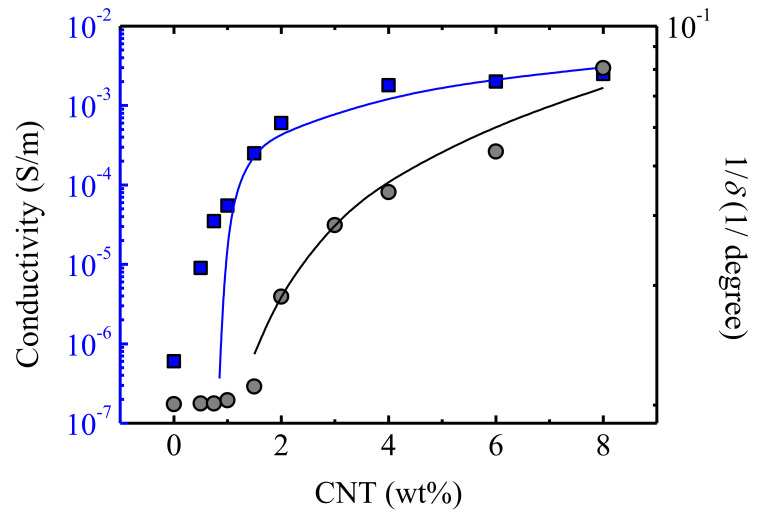
Conductivity and the inverse of the phase angle as a function of the CNT concentration measured at the same Table. 100 °C, i.e., in the molten state. The lines correspond to Equation (1) (see text).

The conductivity measured at the lowest frequency, i.e., 20 Hz, was taken as equivalent to the DC conductivity value. This parameter is plotted in Figure 7 as a function of the CNT content, and we observed an increase of the conductivity with the content of CNT. For comparison with the rheological data, results in the molten state (*T* = 100 °C) were considered.

Applying the percolation theory equation (Equation (1)) with *X* = σ, an electrical percolation threshold of *ϕ_c_* = 0.85 wt% CNT was obtained, with an exponent of *t* = 1. This electrical percolation threshold was similar to that obtained by Koerner et al. [29] for PU/CNT nanocomposites; however, Kim et al. [41] reported a considerably lower value, namely 0.0089 vol%. These differences arose from the CNT type employed, the method used to prepare the nanocomposites and the nature of the polyurethane matrix. Our results, which were obtained in the molten state, were different to the solid state results that have been generally reported in the literature.

With the aim of comparing both the rheological percolation and the electrical percolation, in Figure 7, the inverse of *δ* and the conductivity, both measured at *T* = 100 °C, are plotted as a function of the CNT concentration. The onset of the conductivity enhancement shifted to lower concentrations, as compared to the increase of the inverse of the phase angle, reflecting the lower percolation value obtained through the electrical conductivity measurements (*ϕ_c_* = 0.85 wt% CNT achieved by the electrical conductivity face to *ϕ_c_* = 1 wt% CNT obtained by rheology).

This is in contradiction with literature results that reported a rheological percolation that was lower than electrical percolation [42]. However, in most of the works, the comparison was erroneously made, as the conductivity data were taken in the solid state, and the rheological results were taken in the molten state. In other works, rheological percolation has been observed but not electrical conductivity due to the loss of the effectiveness of nanoparticles as a conducting material [43].

To our knowledge, only two works actually compared electrical percolation obtained in the molten state to rheological percolation obtained, necessarily, in the molten state, with opposing results, namely a lower electrical percolation for PU/CNT nanocomposites [12] and similar percolation thresholds in the case of PU/graphene [44]. Certainly, a comparative study of both types of percolation has not been carried out in depth so far. To develop an electric network, a direct contact between conductive nanoparticles is not strictly necessary, since an electron hopping/tunnelling mechanism [42,45,46,47,48] can take place. However, the restriction of mobility and subsequent formation of a three dimensional network requires topological polymer/CNT interactions. This would explain the observed lower value obtained for the electrical percolation threshold in our case.

### 3.4. Joule Heating Effect

When an electric current is applied to a semiconductor material, heat is generated; this effect is known as the Joule effect and the power of the heating produced is given by the following equation:

(2) $$P=I^{2}\times R$$

where *I* is the electric current intensity, and *R* is the resistance.

**Figure 8:**
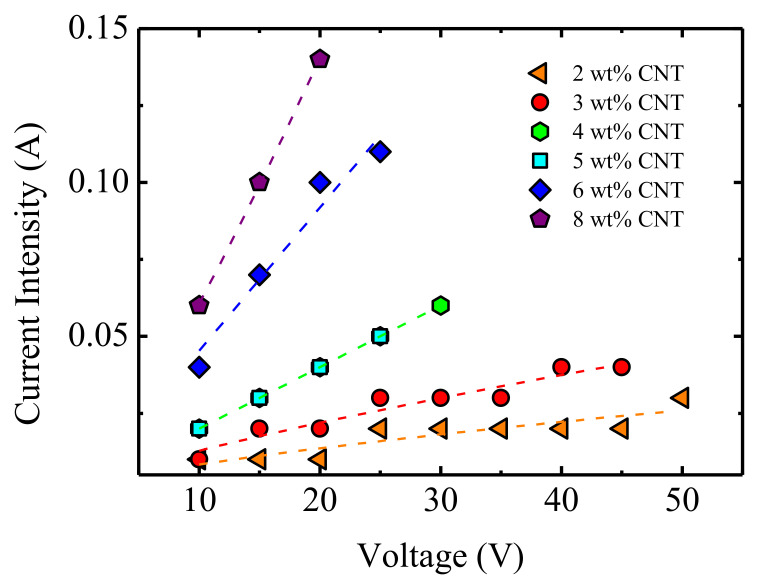
The electric current intensity of the PU/CNT nanocomposites of different compositions as a function of the applied voltage. Lines correspond to a power type equation with the following exponent values (from 2 wt% CNT% to 8 wt% CNT): 0.67; 0.86; 1; 1; 1.14; and 1.22.

During heating effect experiments, the intensity of the current generated when applying a voltage between 10 and 50 V was measured, providing results as shown in Figure 8. When increasing the CNT concentration, an enhancement of the current intensity was observed, as well as an increase in the *I*–*V* slope. For the samples with the lowest CNT concentration (2 and 3 wt%) the sensitivity of the equipment was not sufficient to provide a better resolution, and this resulted in jumps from one value to the next. The data shown in the figure are fitted to a power type equation, *I* = AV^b^. For nanocomposites with filler concentrations lower than the percolation threshold, a non-linear relationship between *I*–*V* was expected (non-Ohmic behavior) [16], whereas for concentrations above the percolation threshold, an Ohmic behavior was predicted. This is explained assuming an electron hopping/tunnelling effect below percolation and direct contact between CNTs, added to tunnelling for above percolation.

For 4 and 5 wt% CNT nanocomposites, the exponent was practically equal to 1, which indicates that there was a linear relationship between the current and applied voltage; therefore, the nanocomposites demonstrated an Ohmic behavior. This analysis is similar to that of Nayak et al. [49] regarding polyimide (PI)/multiwall carbon nanotubes, one of the very scarce papers published on the subject. However, for 6 and 8 wt% CNT nanocomposites, the exponent was above 1, which reflects a non-Ohmic behavior. This positive deviation from linearity in the *I*–*V* plots arises from the Joule heating effect of the sample, since the increase of the temperature reduces the resistivity of the material, as was remarked by Moriche et al. [50].

**Figure 9:**
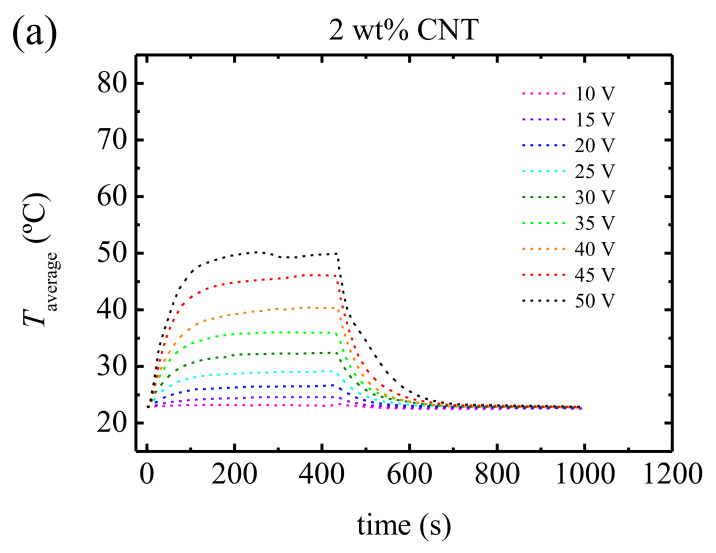

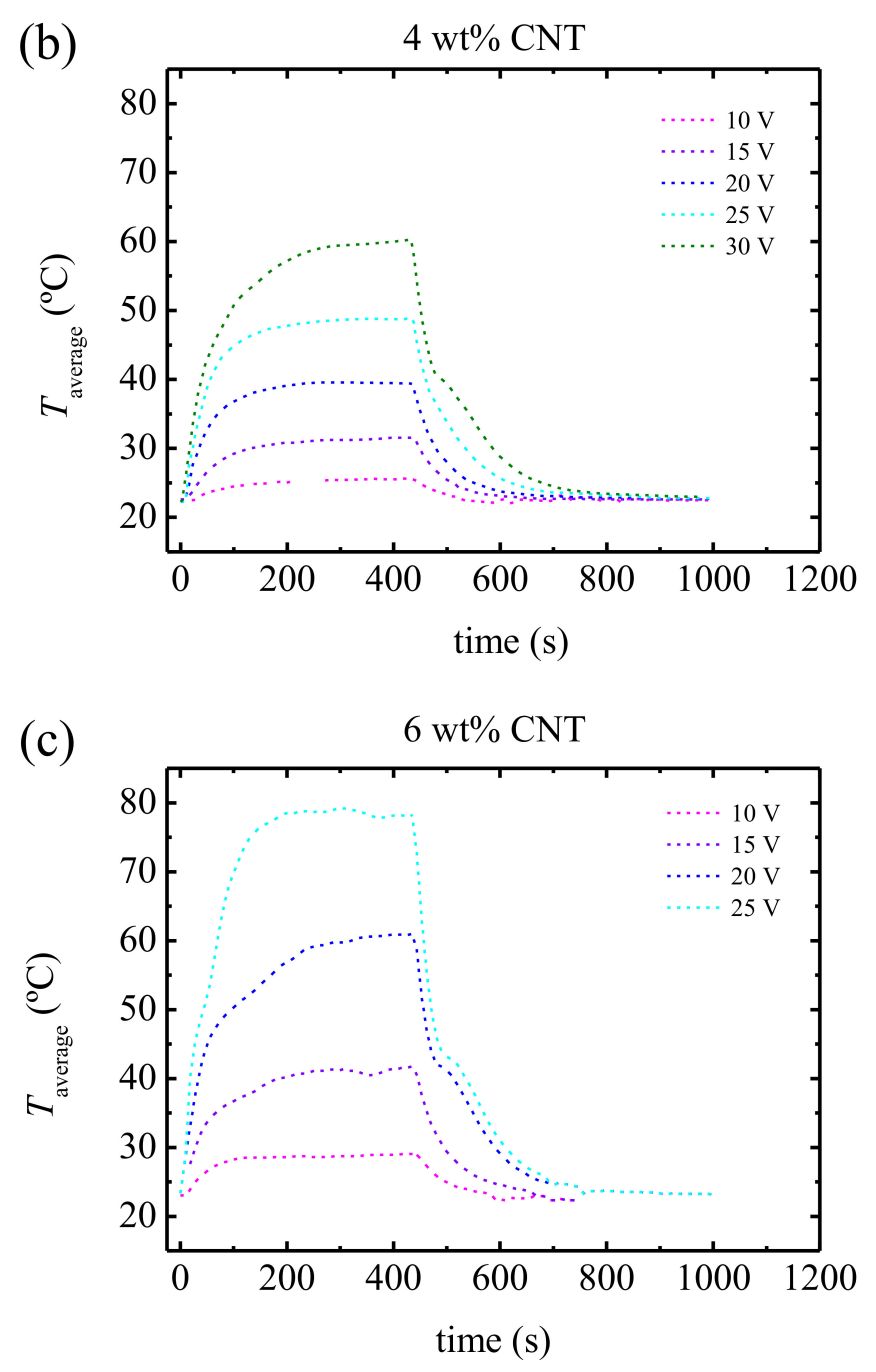

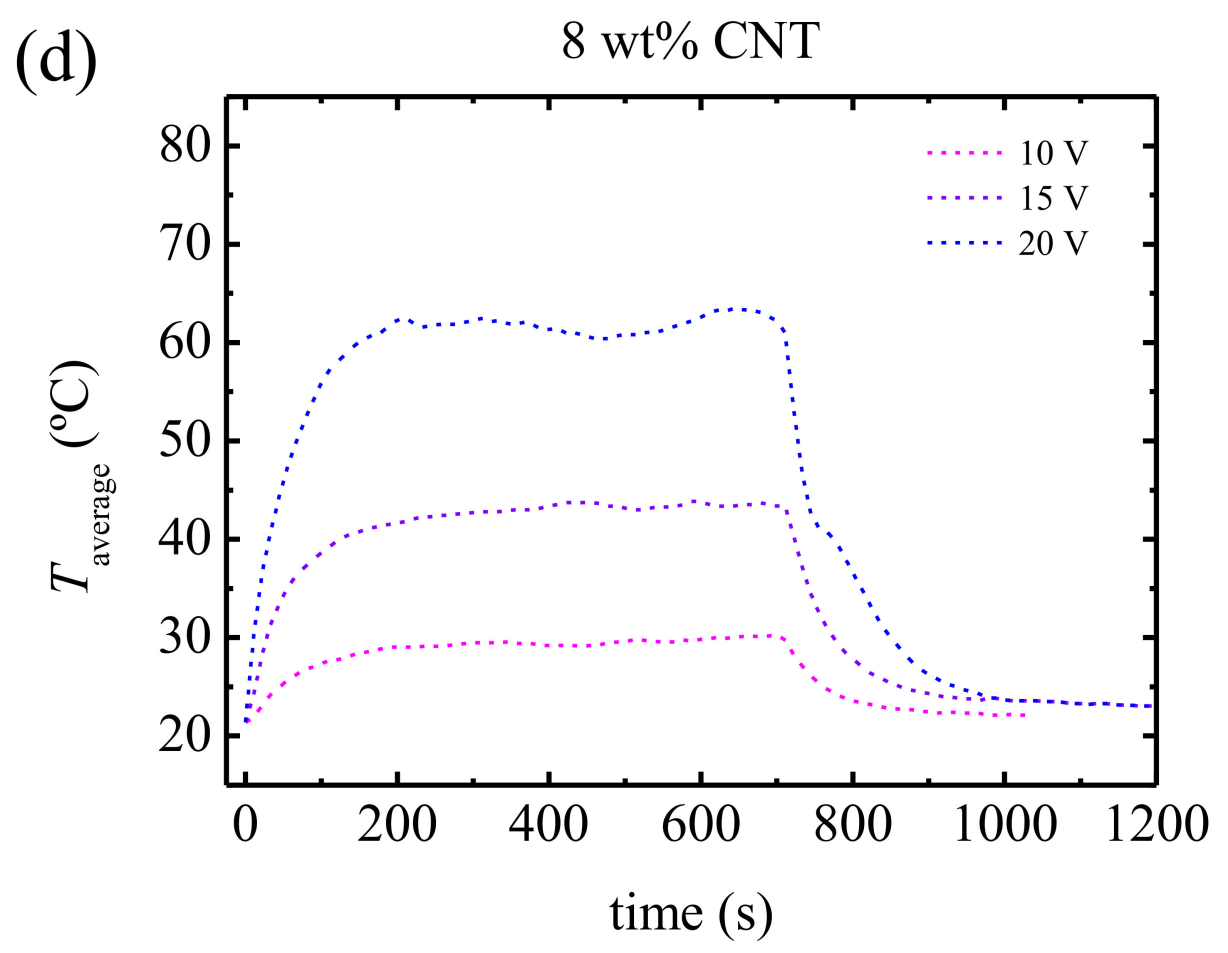
Temperature as a function of time applying and removing the indicated electrical voltages for (**a**) 2, (**b**) 4, (**c**) 6 and (**d**) 8 wt% CNT content (see Supporting Information).

The Joule heating effect has been studied measuring the surface temperature increases of the samples as a function of time for different applied voltages. In Figure 9, the results for some of the nanocomposites studied in this work are shown as an example. The measurements were performed applying, first, a low voltage and then increasing it subsequently. After applying the current with the highest voltage, the lowest voltage was applied again (10 V) to check whether the sample evolved with the different current cycles. We observed that the results were the same as the first measurement. Three zones were distinguished: A fast increase of temperature when the voltage was applied, followed by a steady state, and then a rapid temperature reduction when the voltage was removed. Each zone is fitted to the following equations, respectively: [13,19,51].

(3) $$\mathrm{Heating}\;\mathrm{process}:\;T_{t}=\left(T_{max}-T_{0}\right)\left(1-e^{-t/\tau_{h}}\right)+T_{0}$$

(4) $$\mathrm{Steady}\;\mathrm{state}:\;h_{r+c}=\frac{I_{c}V_{0}}{T_{m}-T_{0}}$$

(5) $$\mathrm{Cooling}\;\mathrm{process}:\;T_{t}=\left(T_{max}-T_{0}\right)\left(e^{-t/\tau_{c}}\right)+T_{0}$$

where *T*_t_ is the temperature at each time, *T*_max_ is the maximum temperature, *T*_0_ is the initial temperature, *τ*_h_ is the characteristic time during heating, *τ*_c_ is the characteristic time during cooling, *h*_r+c_ is the heat transferred by radiation and convection, *I*_c_ is the electric current, and *V*_0_ is the applied voltage.

Analyzing the fitting parameters of these equations (see Tables S1–S6 in the Supplementary Material), in general terms, we observed that increasing the voltage led to an increase of the maximum temperature, a reduction of both, *τ*_h_ and *τ*_c_, an increase of the thermal resistance, *R*_th_ = ∆T⁄P, and a reduction of the specific heat capacity, C_p_ = τ/(*R*_th_ m). The reduction of the respective characteristic times for heating and cooling increasing the voltage is in line with previous results reported for poly(propylene)/CNT and poly(ethylene)/CNT nanocomposites [52].

In Table 1, the maximum temperature values, *T*_max_, at the different applied voltages for each CNT concentration are presented. For 6 wt% CNT, applying a voltage of 25 V produced a temperature increase with respect to room temperature (ΔT = *T*_max_ − 25 °C) of 55 °C, which is an encouraging result in view of the literature data. Recently, Yum et al. [53] studied a PU/CNT nanocomposite coating, reaching an increase, ΔT = *T*_max_ − 25 °C, of only 15 °C when applying a voltage of 50 V to a 7% CNT concentration sample. In our previous study with PP/CNT [52], an increase of 90 °C was reached with a voltage of 20 V. A remarkable result was reported by Willocq et al. [54] for a poly(ether-urethane)/carbon nanotube nanocomposite observing an increase of 50 °C when applying a voltage of 25 V with a concentration of only 2 wt% CNT. These differences arise from the dissimilar casuistry of the polymer matrix and CNT features and the nanocomposite elaboration method.

In our case, the heating effect, expressed as the maximum reached temperature, *T*_max_, was enhanced as the CNTs increased, even above the electrical percolation threshold. This contradicts the decreasing heating trend noted by Prolongo et al. [16] for CNT concentrations above percolation, observed in their epoxy/CNT nanocomposites. According to these authors, the increase of the CNT concentration in the composites above the percolation threshold enhanced the contact between electrical conductor nanotubes, decreasing the heating due to the Joule effect.

Our results are compatible with this hypothesis, assuming that, in our nanocomposites, electron hopping mechanisms [42,45,46,47,48], but not necessarily direct contact with CNTs, take place. The lower electrical percolation threshold compared with the rheological threshold, mentioned above, would support this assumption.

**Figure 10:**
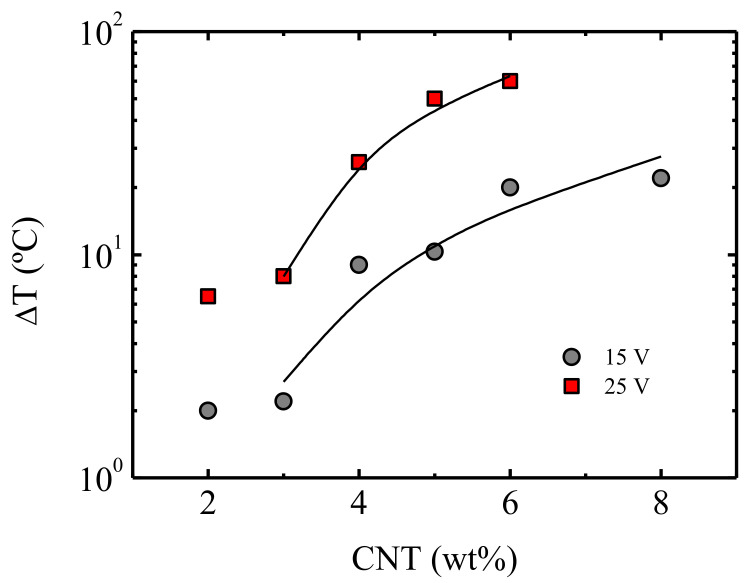
The maximum temperature increase ΔT = T_max_ − T as a function of the CNT content for applied voltages between 10 and 25 V. The data are fitted to Equation (1) with *X* = ΔT (see text).

In Figure 10, the reached maximum temperature increase ΔT = *T*_max_ − *T*, for two applied voltages is plotted as a function of the CNT concentration. The results are fitted to the equation of the statistical percolation theory Equation (1), considering that, in this case, the physical property under evaluation is *X* = ΔT. This allows us to introduce a percolation threshold that stands for the Joule heating effect.

The corresponding fitting values are *ϕ_c_* = 2 wt% CNT and *t* = 1.2 for 15 Volts as well as *ϕ_c_* = 2.5 wt% CNT and *t* = 0.95 for 25 Volts. Comparing the respective rheological and electrical percolation threshold values (discussed above) to the newly defined heating percolation threshold, we observed that the higher value corresponded to the latter. A higher threshold for efficient Joule heating over electrical conductivity was also reported by Jeong et al. [19] in the case of epoxy/graphene nanocomposite films, in the sole published paper on the matter. This result is logical recalling that the Joule heating effect requires energy exchange between the accelerated electrons and the atomic ions that release the extra energy as heat.

**Figure 11:**
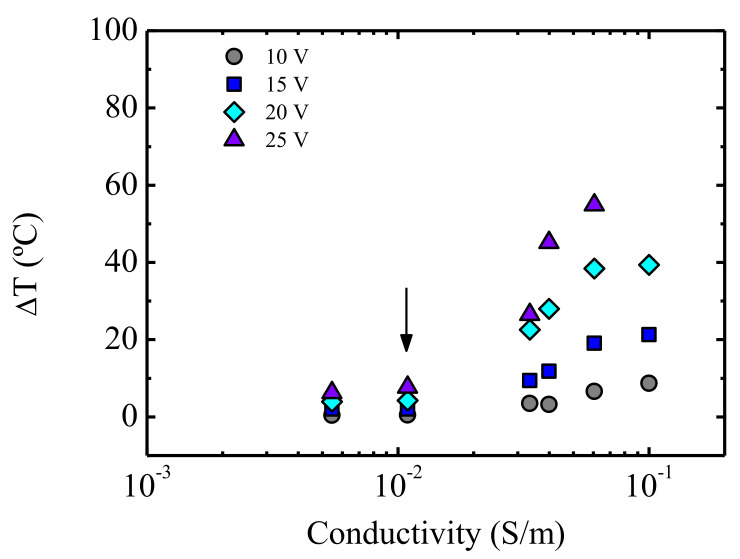
Temperature increase as a function of conductivity for PU/CNT nanocomposites. The data correspond to different CNT concentrations, since each nanocomposite is identified by its conductivity. See the text for the meaning of the arrow.

With the goal of linking the temperature increase (ΔT, obtained at different voltages) to an intrinsic property, such as the DC conductivity (*σ*, measured by dielectric experiments at *T* = 25 °C), in Figure 11 ΔT is plotted against *σ*. A jump of the slope of ΔT was observed (marked with an arrow) at the conductivity of *σ* = 0.012 S m^−1^. The shortage of literature data correlating the DC conductivity to the Joule heating effect impedes posing any hypothesis regarding the existence of a critical conductivity value that triggers heating, provoking a jump in temperature.

ΔT versus conductivity plots can be very useful to investigate the practical advantages of polymer systems that provide heat within the field of stimuli responsive materials. A nanocomposite with an adequate conductivity can be selected depending on the required increase of temperature and a suitable voltage.

The main practical application of our PU/CNT nanocomposites is their use as hot melt adhesives. After melting, the surfaces to be bonded weld and, on cooling, the nanocomposites solidify through crystallization. The presence of CNTs accelerates the crystallization, thus reducing the welding time [11]. For the purposes that are explained below, the melting temperature, *T*_m_, of the nanocomposites was in the range of 54–56 °C [11].

These temperatures were surpassed by several of our PU/CNT nanocomposites using the Joule effect when applying voltages as typically employed in our work. For instance, a temperature of *T* = 59 °C was reached when a voltage of 45 V was applied to a nanocomposite that contained only a concentration of 3 wt% CNTs; also, *T* = 62 °C was reached with a voltage of 20 V applied to a nanocomposite with a concentration of 8 wt% CNT. With these results, a stimulus responsive adhesive can be envisaged. Considering the rapid transitory responses during the application and cease of electric tension (Figure 9), a reversible hot melt adhesive can be obtained by connecting a PU/CNT nanocomposite to a low voltage device (below 50 V).

Taking advance of the Joule effect, the nanocomposite located between two substrates was heated and melted, bringing about the necessary tack to immediate adhesion. Removing the applied tension brought the nanocomposite rapidly back to room temperature, allowing the crystallization process to begin. Once the substrates are welded, they can be unglued by applying an electrical tension to surpass the melting temperature of the PU/CNT nanocomposite adhesive. Certain devices, which are out of the scope of this paper, can be designed based on this stimulus–response behavior.

## 4. Conclusions

Thermal analysis showed that the presence of CNTs did not increase the melting temperature, which is advantageous for Joule heating applications, whereas this increased the nucleation density, shifting the crystallization temperature to higher temperatures. A new method to determine the rheological percolation threshold was proposed.

The concentration of nanoparticles necessary to form a three-dimensional percolated network was evaluated by the analysis of a delta phase peak observed in Mavridish–Shroff or Booij-Palmen plots (*δ* versus log G*), which represent the efficiency of CNT particles to hinder chain motions. The intensity of the peak and the associated relaxation time were analyzed; the equation of the statistical percolation theory provided the same percolation threshold for both: *ϕ_c_* = 1 wt% CNT. The electrical percolation threshold obtained from the conductivity data was observed to be lower, *ϕ_c_* = 0.85 wt% CNT, which is explained by assuming that a hopping/tunnelling effect takes place.

The analysis of the Joule heating effect revealed considerable temperature increases with respect to room temperature. For instance a temperature of 60 °C was obtained indistinctly when a voltage of 45 V was applied to a nanocomposite that contained only a concentration of 3 wt% CNTs, whereas a voltage of 20 V was applied to a nanocomposite of a concentration of 8 wt% CNT. Through analyzing the variation of the temperature increase, ΔT, with the concentration of CNTs in light of the equation of the statistical percolation theory, a percolation threshold that stands for the Joule heating effect was proposed. The value of this newly defined threshold is *ϕ_c_* = 2 wt% CNT.

The coupled study of the electrical conductivity obtained by dielectric measurements and the Joule effect in non-Ohmnic conditions led to the establishment of a critical conductivity value that triggered a heating effect, giving rise to a jump in temperature. The results were compared with those of a series of PP/CNT nanocomposites. Plots of ΔT as a function of electrical conductivity are presented to display the demanded temperature increase of a nanocomposite in view of its electrical conductivity.

Considering that our PU/CNT nanocomposites are used as hot melt adhesives, we can take advantage of the Joule effect to develop stimulus-responsive glues. The melting temperatures of our nanocomposites are in the range of 54–56 °C; therefore, they can be surpassed by applying low/moderate voltages. With this premise, a nanocomposite located between two substrates can be heated and melted to produce tackiness; in removing the applied tension, the nanocomposite is cooled allowing crystallization and welding to occur. The electrically stimulated process is reversible, since ungluing can be triggered by applying the electrical tension once again.

## Figures and Tables

**Table 1:** The *T*_max_ reached by the nanocomposites when applying different voltages.

Applied Voltage (V)	*T*_max_ (°C)					
	2 wt%	3 wt%	4 wt%	5 wt%	6 wt%	8 wt%
10	23.4	21.8	25.6	27.4	29.0	30.2
15	24.6	23.6	31.5	35.0	41.7	43.3
20	26.5	25.8	39.5	50.3	60.9	62.1
25	29.0	29.3	48.9	67.8	78.4	/
30	32.3	34.7	60.2	/	/	/
35	36.0	41.3	/	/	/	/
40	40.3	52.8	/	/	/	/
45	46.1	59.5	/	/	/	/
50	49.8	/	/	/	/	/

## Data Availability

Not applicable.

## Supplementary Materials

The following are available online at https://www.mdpi.com/2073-4360/13/6/950/s1, Tables S1–S6: Parameters obtained employing Equations (3)–(5) for different PU/CNT nanocomposites: characteristic times, τ_h_ and τ_c_, thermal resistance, *R*_th_ = ∆T⁄P and specific heat capacity, *C_p_* = τ/(R_th_ m).
