# Supplementary material for: Matching Rheology, Conductivity and Joule Effect in PU/CNT Nanocomposites
Source: Polymers (Basel). 2021 Mar 19;13(6):950. doi: 10.3390/polym13060950 (PMC8003351; doi:10.3390/polym13060950)
Supplement: Supplementary file 1 [file polymers-13-00950-s001.pdf]

Supporting Information

Table 1. Parameters obtained employing Equations 3-5 for 2 wt% CNT: characteristic times, τ_h and τ_c, thermal resistance, R_{th}=ΔT/P, and specific heat capacity, C_p=τ/(R_{th} m).

**Voltage**	h_{r+c}	τ_h	τ_c	R_{th}	C_p
V	mW°C^{-1}	s	s	°CW^{-1}	Jg^{-1}K^{-1}
10	152.4	9.3	58.2	4.5	0.29
15	51.2	80.3	54.9	13.4	3.63
20	35.0	70.3	56.7	19.6	2.17
25	54.7	60.6	55.3	12.5	2.93
30	43.4	59.9	60.4	15.8	2.30
35	37.4	54.0	52.7	18.3	1.79
40	30.4	62.2	60.6	22.6	1.67
45	26.4	58.7	61.6	26.0	1.37
50	37.6	53.6	78.7	18.2	1.78

Table 2. Parameters obtained employing Equations 3-5 for 3 wt% CNT: characteristic times, τ_h and τ_c, thermal resistance, R_{th}=ΔT/P, and specific heat capacity, C_p=τ/(R_{th} m).

**Voltage**	h_{r+c}	τ_h	τ_c	R_{th}	C_p
V	mW°C^{-1}	s	s	°CW^{-1}	Jg^{-1}K^{-1}
10	212.8	/	/	4.7	/
15	110.3	112.0	46.3	9.1	6.6
20	94.1	67.2	56.6	10.6	4.9
25	81.2	70.7	50.0	12.3	3.7
30	70.5	64.8	60.4	14.2	3.2
35	53.7	72.8	46.4	18.6	2.2
40	52.6	105.2	53.8	19.0	2.4
45	48.1	42.6	53.8	20.8	2.1

Table 3. Parameters obtained employing Equations 3-5 for 4 wt% CNT: characteristic times, τ_h and τ_c, thermal resistance, R_{th}=ΔT/P, and specific heat capacity, C_p=τ/(R_{th} m).

**Voltage**	h_{r+c}	τ_h	τ_c	R_{th}	C_p
V	mW°C^{-1}	s	s	°CW^{-1}	Jg^{-1}K^{-1}
10	35.7	81.8	80.3	17.3	3.1
15	29.5	73.0	63.2	21.0	2.3
20	21.9	54.3	65.1	28.2	1.2
25	29.0	53.9	81.7	21.2	1.6
30	29.5	70.9	22.0	20.9	2.2

Table 4. Parameters obtained employing Equations 3-5 for 5 wt% CNT: characteristic times, τ_h and τ_c, thermal resistance, R_{th}=ΔT/P, and specific heat capacity, C_p=τ/(R_{th} m).

**Voltage**	h_{r+c}	τ_h	τ_c	R_{th}	C_p
V	mW°C^{-1}	s	s	°CW^{-1}	Jg^{-1}K^{-1}
10	37.9	34.8	38.9	16.3	1.6
15	23.5	46.7	53.2	26.2	1.3
20	17.6	49.3	72.3	35.0	1.0
25	17.1	52.2	69.6	36.1	1.1

Table 5. Parameters obtained employing Equations 3-5 for 6 wt% CNT: characteristic times, τ_h and τ_c, thermal resistance, R_{th}=ΔT/P, and specific heat capacity, $C_p=\tau/(R_{th}\ m)$.

**Voltage**	h_{r+c}	τ_h	τ_c	R_{th}	C_p
V	$mW°C^{-1}$	s	s	$°CW^{-1}$	$Jg^{-1}K^{-1}$
5	106.4	109.2	109.2	5.8	13.4
10	37.5	50.4	56.3	16.5	2.2
15	34.0	72.0	62.8	18.1	2.8
20	32.2	75.1	83.4	19.2	2.8
25	30.9	56.1	72.3	20.0	2.0

Table 6. Parameters obtained employing Equations 3-5 for 8 wt% CNT: characteristic times, τ_h and τ_c, thermal resistance, R_{th}=ΔT/P, and specific heat capacity, $C_p=\tau/(R_{th}\ m)$.

**Voltage**	h_{r+c}	τ_h	τ_c	R_{th}	C_p
V	$mW°C^{-1}$	s	s	$°CW^{-1}$	$Jg^{-1}K^{-1}$
5	173.6	10.3	/	3.2	2.0
10	38.2	97.1	55.5	14.6	4.1
15	39.1	64.8	75.8	14.2	2.8
20	39.5	52.8	76.5	14.1	2.3
